# An enteric ultrastructural surface atlas of the model insect *Manducasexta*

**DOI:** 10.1016/j.isci.2024.109410

**Published:** 2024-03-04

**Authors:** Anton G. Windfelder, Jessica Steinbart, Leonie Graser, Jan Scherberich, Gabriele A. Krombach, Andreas Vilcinskas

**Affiliations:** 1 Branch for Bioresources, Fraunhofer Institute for Molecular Biology and Applied Ecology IME, Giessen, Germany; 2 Experimental Radiology, Department of Diagnostic and Interventional Radiology, University-Hospital Giessen, Justus Liebig University Giessen, Giessen, Germany; 3 Department of Diagnostic and Interventional Radiology, University-Hospital Giessen, Giessen, Germany; 4 Institute for Insect Biotechnology, Department of Applied Entomology, Justus Liebig University Giessen, Giessen, Germany

**Keywords:** Microscopic anatomy, Biological sciences, Entomology

## Abstract

The tobacco hornworm is a laboratory model that is particularly suitable for analyzing gut inflammation, but a physiological reference standard is currently unavailable. Here, we present a surface atlas of the healthy hornworm gut generated by scanning electron microscopy and nano-computed tomography. This comprehensive overview of the gut surface reveals morphological differences between the anterior, middle, and posterior midgut, allowing the screening of aberrant gut phenotypes while accommodating normal physiological variations. We estimated a total resorptive midgut surface of 0.42 m^2^ for L5d6 larvae, revealing its remarkable size. Our data will support allometric scaling and dose conversion from *Manduca sexta* to mammals in preclinical research, embracing the 3R principles. We also observed non-uniform gut colonization by enterococci, characterized by dense biofilms in the pyloric cone and downstream of the pylorus associated with pore and spine structures in the hindgut intima, indicating a putative immunosurveillance function in the lepidopteran hindgut.

## Highlights

• This is a systematic surface atlas of the healthy hornworm gut surface
• We estimated a total resorptive midgut surface as 0.42 m^2^ for L5d6 larvae
• We observed dense biofilms of enterococci in the pyloric cone
• This work supports allometric scaling and dose conversion according to the 3Rs

## Graphical abstract

**Figure:**
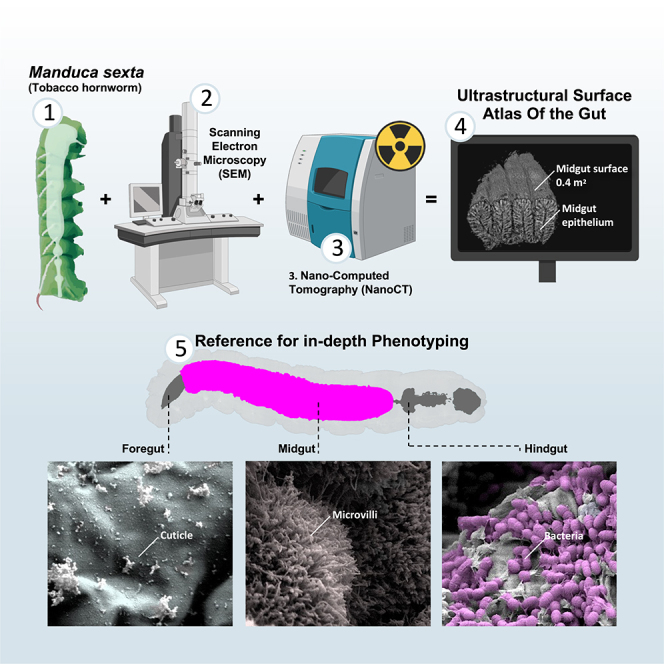

## Introduction

Preclinical research has long been reliant on small mammals such as mice, but heightened ethical considerations have prompted a shift toward the 3R principles (replacement, reduction, and refinement) in regulations governing animal experiments and research funding.^1^^,^^2^ In this evolving context, insect larvae have emerged as promising alternative *in vivo* animal model systems, not only because certain physiological aspects are remarkably similar to humans but also because they can be bred and reared in large numbers at very low costs.^3^^,^^4^

The tobacco hornworm (*Manduca sexta*) is an insect model organism with many advantages for preclinical research, including a fully mapped genome,^5^^,^^6^ methylome,^7^ and the availability of specific monoclonal antibodies.^8^ The large size of *M. sexta* larvae, regularly exceeding 10 g, is also ideal for experiments in biochemistry,^9^ developmental biology,^10^ immunology,^9^^,^^11^^,^^12^ epigenetics,^7^ morphology,^13^ neurobiology,^14^ and gut physiology.^15^ There is a high degree of evolutionary conservation between the *M. sexta* and human gut, including notable similarities in enteric epithelial structures and innate immunity.^13^^,^^16^

Based on these advantages, we previously established *M. sexta* as a non-vertebrate model of gut inflammation that adheres to the 3R principles.^16^ We have used medical imaging modalities such as computed tomography (CT),^13^ magnetic resonance imaging (MRI),^17^ and positron emission tomography (PET),^18^ exploiting the large size of *M. sexta* larvae for the image-guided high-throughput screening of aberrant gut phenotypes, including screening for new effectors and inhibitors of gut inflammation, pesticides, antibiotics, and host–pathogen interactions,^16^ as well as the identification of new contrast agents for medical imaging.^19^

Here, we present an ultrastructural surface atlas of the gut generated by scanning electron microscopy (SEM), allowing the rapid and detailed phenotyping of gut-associated pathologies (infection or inflammation) that can trigger epithelial erosion, alter plasma membrane dynamics and change enterocyte morphology.^16^^,^^20^^,^^21^^,^^22^^,^^23^^,^^24^^,^^25^ However, there are anatomical and physiological differences between parts of the gut *of M. sexta* that are not pathological, so it is important to have a standard reference in healthy insects that can be used for comparative purposes. This study, therefore, builds on previous studies of *M. sexta* gut morphology to provide a comprehensive reference atlas.^10^^,^^16^^,^^26^^,^^27^^,^^28^^,^^29^ In particular, this study complements our previously published quantitative three-dimensional micro-tomographic gut atlas of *M. sexta*.^13^

We also considered the transition from murine to insect models by calculating the total resorptive midgut surface area, allowing allometric scaling for oral dose conversion, using our previously established μCT method and a clinical contrast agent. This was sufficient for the overall volume calculations and revealed the most prominent gut folds, but to quantify the deep villus-like gut folds, we combined an advanced nano- and micro-computed tomographic (CT) approach with SEM. Previously, we revealed a striking similarity in gut volume between *M. sexta* and murine models.^13^ Here, we determined the gut surface area of last-instar (L5d6) larvae compared to mice, for a corresponding oral dose conversions.^30^ Finally, having previously characterized the *M. sexta* larval microbiome and revealed a bacterial community dominated by two species of enterococci,^16^ we investigated the spatial pattern of bacterial colonization in the gut, mapping areas of high bacterial density. Our main findings are animated in Videos S1, S2, S3, S4, S5, S6, S7, S8, S9, and S10, which can be used as teaching and training resources for higher educational institutions and research organizations.

Video S1. Foregut of *M. sexta* A folded intima with a rough surface dominates the cuticle-lined surface of the foregut.

Video S2. Anterior midgut of *M. sexta* A dense peritrophic matrix covers the gut epithelium. Enterocytes show irregular club-shaped microvilli. The cell boundaries are visible.

Video S3. Anterior midgut of *M. sexta* The gut epithelium consists of columnar cells and goblet cells. The irregular club-shaped microvilli gradually approach their standard shape in the anterior midgut towards the middle midgut.

Video S4. Anterior midgut of *M. sexta* with an intact peritrophic matrix Note the bacteria colonizing the peritrophic matrix on the endoperitrophic surface.

Video S5. Middle midgut of *M. sexta* The prominent midgut folds of the gut epithelium are covered by the peritrophic matrix. Microvilli appear under the peritrophic matrix.

Video S6. Posterior midgut of *M. sexta* The enterocytes gently protrude into the posterior gut lumen. The peritrophic matrix can easily detach from the posterior midgut region and is not shown.

Video S7. Hindgut (pyloric cone) of *M. sexta* In sharp contrast to the midgut, the folded intima of the pyloric cone (hindgut) is densely covered with a bacterial biofilm.

Video S8. Hindgut (ileum) of *M. sexta* The folded intima is spiculated and densely populated with bacteria. At higher magnification, pores in the intima become evident.

Video S9. Hindgut (colon) of *M. sexta* The heavily folded intima of the colon lacks spines and bacteria. The intima has spherical imprints (sacculations) at higher magnification and pores are visible.

Video S10. Hindgut (rectum) of *M. sexta* In contrast to the more anterior hindgut parts, the rectum has a smooth intima.

## Results

### Structural organization of the digestive system

In *M. sexta* larvae, like most insects, the digestive system can be divided into three main sections: the foregut (stomodeum) lined with a cuticle (intima), the midgut (mesenteron), and the hindgut (proctodeum), which is also lined with a cuticle (intima).^13^

### Foregut

The intima of the foregut is heavily folded and smooth (Figures S1, S2, and Video S1). Previously, we showed that the intima of the foregut is highly extendable and can form a crop.^13^ The foregut opens into the midgut, marked by the presence of the stomodeal valve, which extends into the midgut lumen.^13^ The surface of the stomodeal valve is similar to that of the foregut (Figure S3). Previously, we reported the volume of the L5d6 foregut as 0.03 ml, with a mean area of 68.57 mm^2^.^13^

### Midgut

Based on its general morphology, the *M. sexta* midgut can be subdivided into the anterior, middle, and posterior midgut.^13^^,^^29^ Previously, we showed that six rudimentary caeca are located at the most anterior part of the anterior midgut.^13^ In this region, we observed elongated cylindrical structures of unknown function in an annular arrangement, which do not occur elsewhere in the intestine (Figure S4). In our previous paper, we documented a midgut volume of 1.4 ml.^13^

### Midgut epithelium and peritrophic matrix

**Figure 1:**
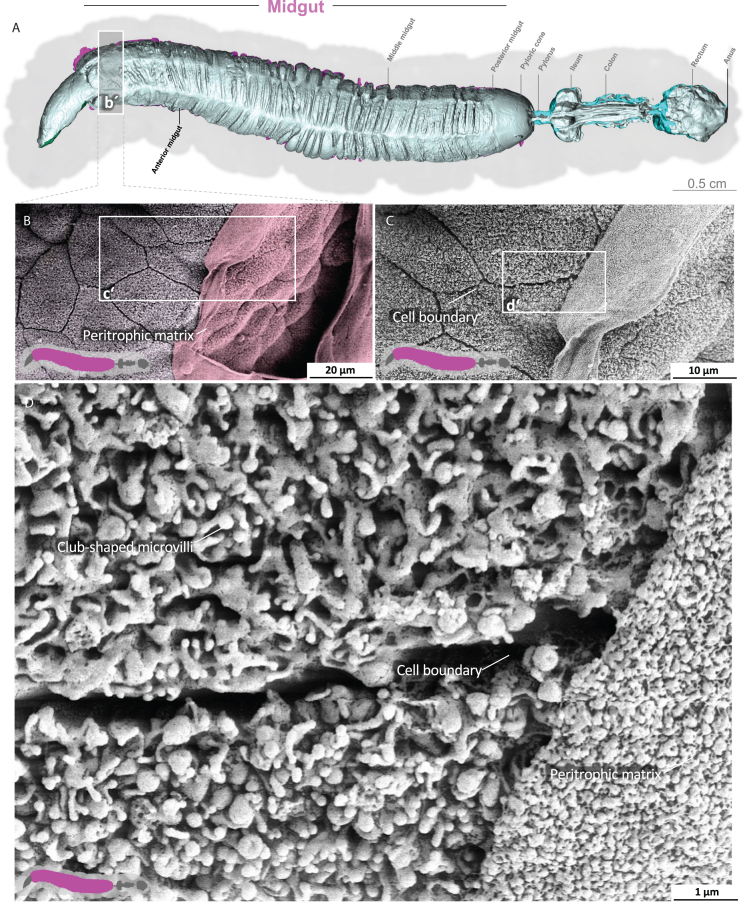
Anterior midgut of *M. sexta* A dense peritrophic matrix covers the gut epithelium. Enterocytes show irregular club-shaped microvilli. The cell boundaries are visible. A micro-tomographic surface overview of the digestive system of *M. sexta* (A) shows the localization (b′) of the SEM insets (B–D). The image in panel (B) is artificially colored to highlight the peritrophic matrix.

**Figure 2:**
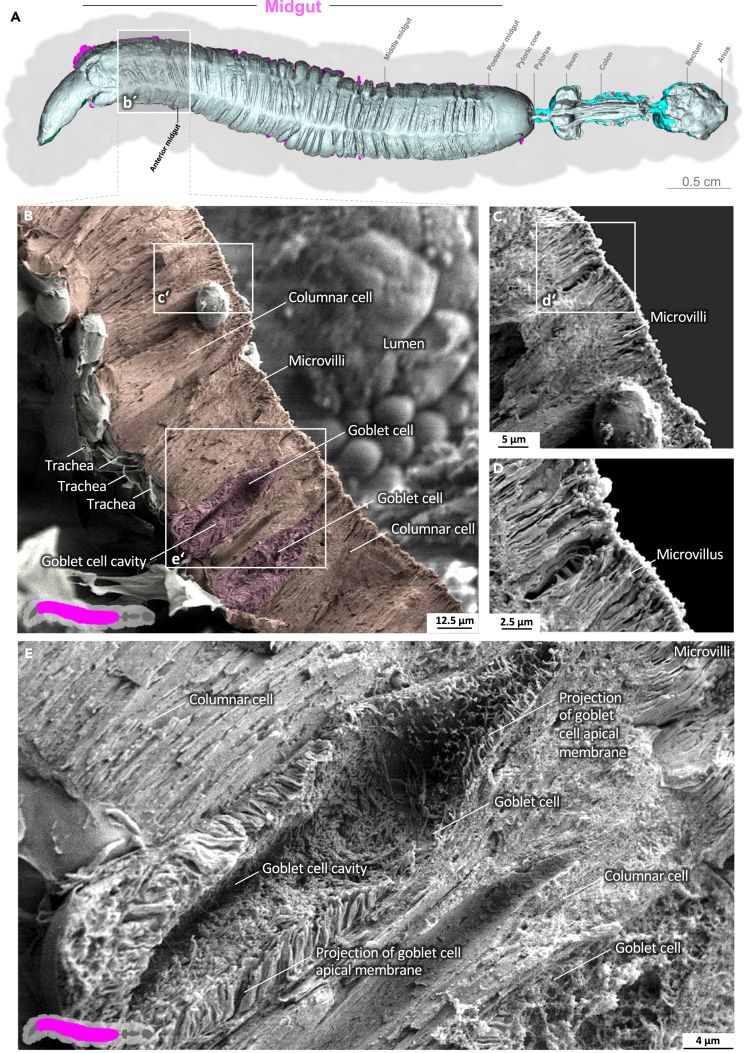
Anterior midgut of *M. sexta* (epithelial cross-section) The gut epithelium consists of columnar cells and goblet cells. The irregular club-shaped microvilli gradually approach their standard shape in the anterior midgut toward the middle midgut (Figure 3). A micro-tomographic surface overview of the digestive system of *M. sexta* (A) shows the localization (b′) of the SEM insets (B–E). The image in panel (B) is artificially colored to highlight the goblet cells.

**Figure 3:**
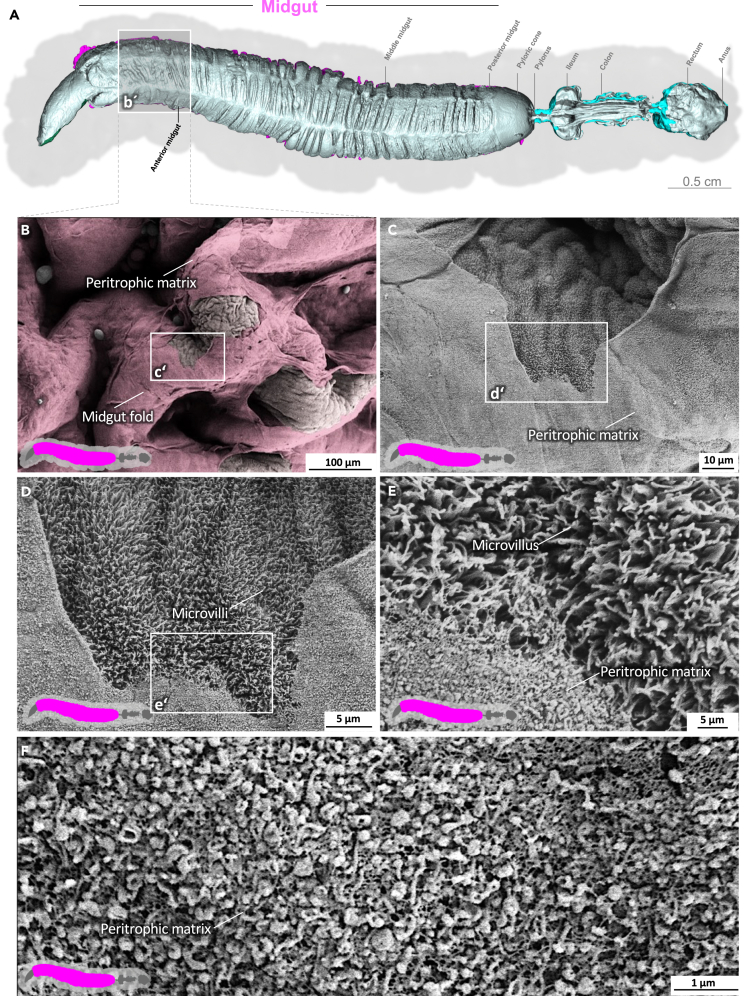
Anterior midgut of *M. sexta* The midgut folds are covered with the peritrophic matrix. The microvilli have reached their standard shape. A micro-tomographic surface overview of the digestive system of *M. sexta* (A) shows the localization (b′) of the SEM insets (B–F). The image in panel (B) is artificially colored to highlight the peritrophic matrix.

**Figure 4:**
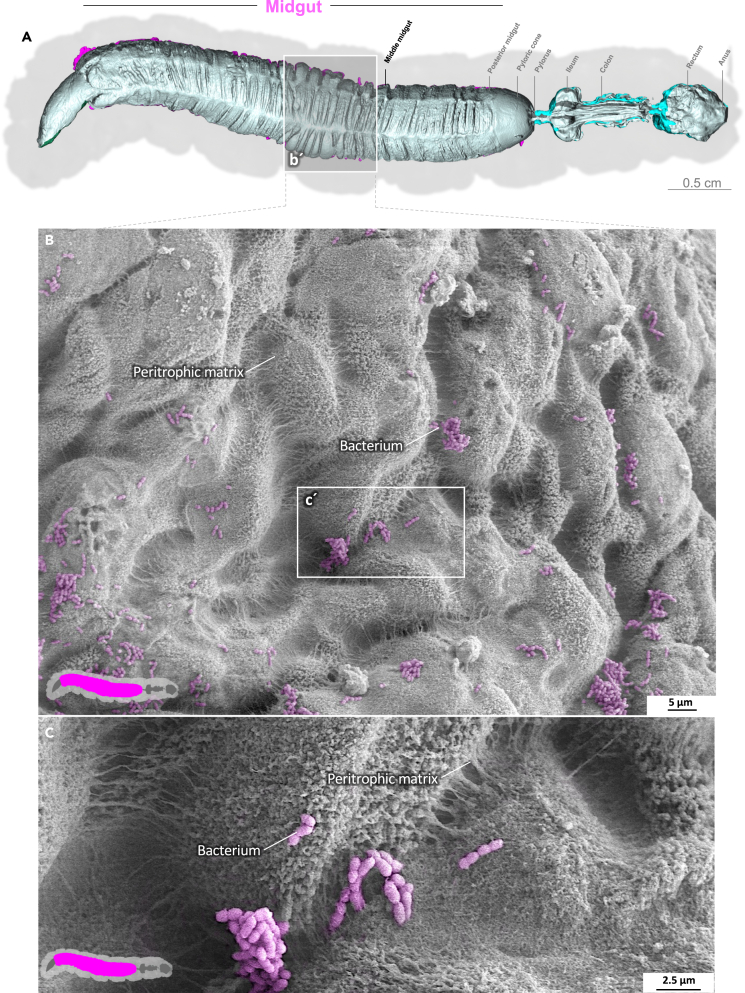
Anterior midgut of *M. sexta* with an intact peritrophic matrix Note the bacteria colonizing the peritrophic matrix on the endoperitrophic surface. A micro-tomographic surface overview of the digestive system of *M. sexta* (A) shows the localization of the SEM inset (B and C).

**Figure 5:**
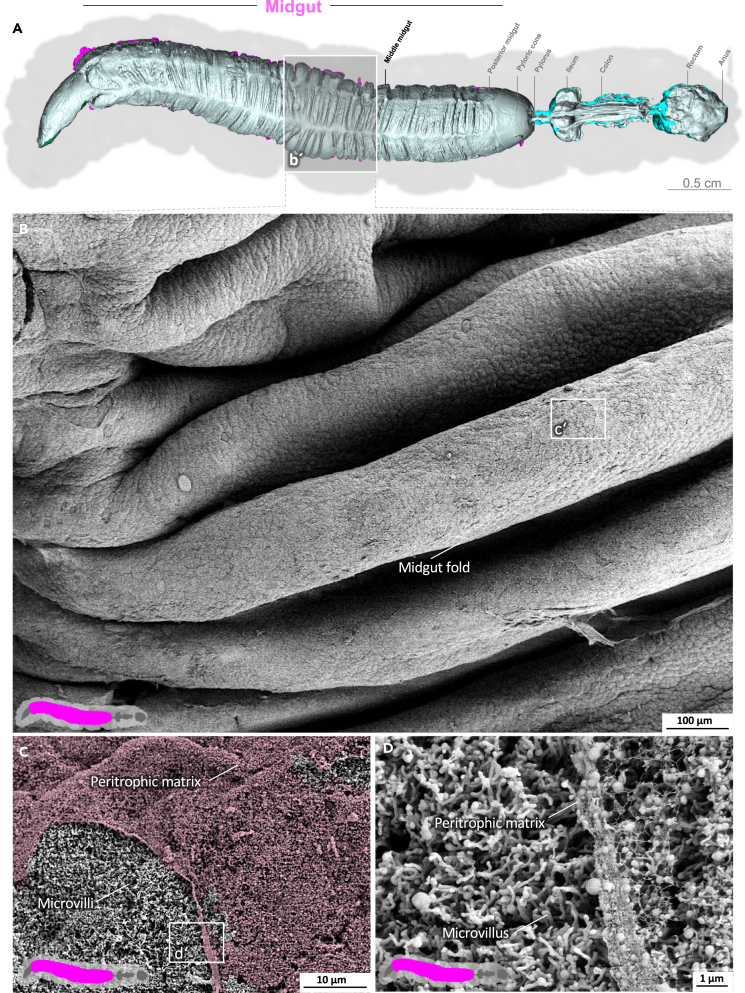
Middle midgut of *M. sexta* The prominent midgut folds of the gut epithelium are covered by the peritrophic matrix. Microvilli appear under the peritrophic matrix. A micro-tomographic surface overview of the digestive system of *M. sexta* (A) shows the localization (b′) of the SEM insets (B–D). The image in panel (C) is artificially colored to highlight the peritrophic matrix.

**Figure 6:**
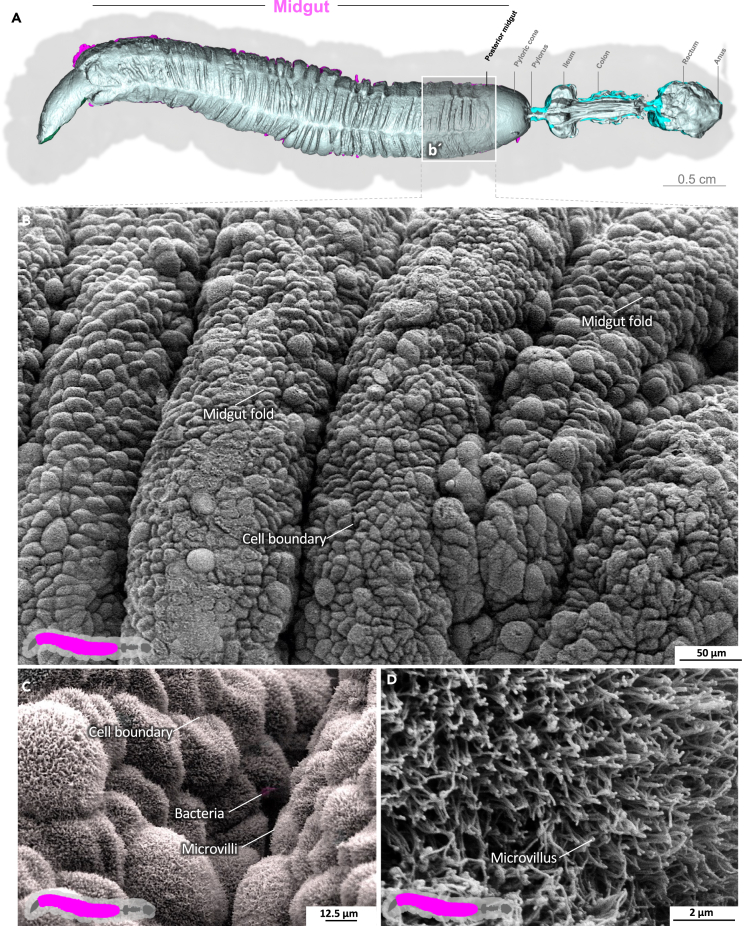
Posterior midgut of *M. sexta* The enterocytes gently protrude into the posterior gut lumen. The peritrophic matrix can easily detach from the posterior midgut region and is not shown. A few bacteria are directly exposed to the microvilli, as shown in panel (C). The image in panel (C) is artificially colored to highlight the bacteria on top of the microvilli. A micro-tomographic surface overview of the digestive system of *M. sexta* (A) shows the localization (b′) of the SEM insets (B–D).

The midgut is the primary site of nutrient resorption. Microvilli are membrane protrusions that increase the surface area for the absorption or secretion in most cells in the midgut. In contrast to the microvilli in the middle and posterior regions, the microvilli in the anterior midgut show extensive thickening and appear as club-shaped microvilli (Figure 1).^29^ The cell boundaries of the enterocytes are visible, and the peritrophic matrix has a felt-like structure, with pronounced nodules (Figure 1D; Video S2). The shape of the microvilli changes gradually in an anterior-to-posterior gradient (Figures 1, 2, and 3). The microvilli in the middle anterior midgut remain condensed and adopt their familiar shape only in the rear anterior midgut (Figure 3; Video S3). The two most common cell types in the gut epithelium, the columnar cells and goblet cells, are visible in cross-sections of the gut epithelium (Figure 2; Video S3). When the peritrophic matrix is completely intact, it covers the entire intestinal epithelium like a cloth. Filaments are visible in the topological canyons of the underlying epithelium (Figure 4; Video S4). The entire peritrophic matrix on the endoperitrophic surface is lightly colonized with bacteria (Figure 4; Video S4). When the peritrophic matrix is partially or completely detached, bacteria are no longer visible (Figures 5, S5, and Video S5), with very few exceptions (Figure 6C). The bacteria are present in the midgut and hindgut. They have an ovoid shape and appear to occur in pairs or chains of different lengths (Figures S7 and Figure 8E). According to our previous characterization of the *M. sexta* microbiome, these bacteria belong to the genus *Enterococcus*.^16^^,^^31^ The enterocytes of the posterior midgut gently protrude into the gut lumen. The peritrophic matrix can easily detach from the posterior midgut region and is not shown (Figure 6; Video S6).

### Estimation of the total surface area of the midgut

**Figure 7:**
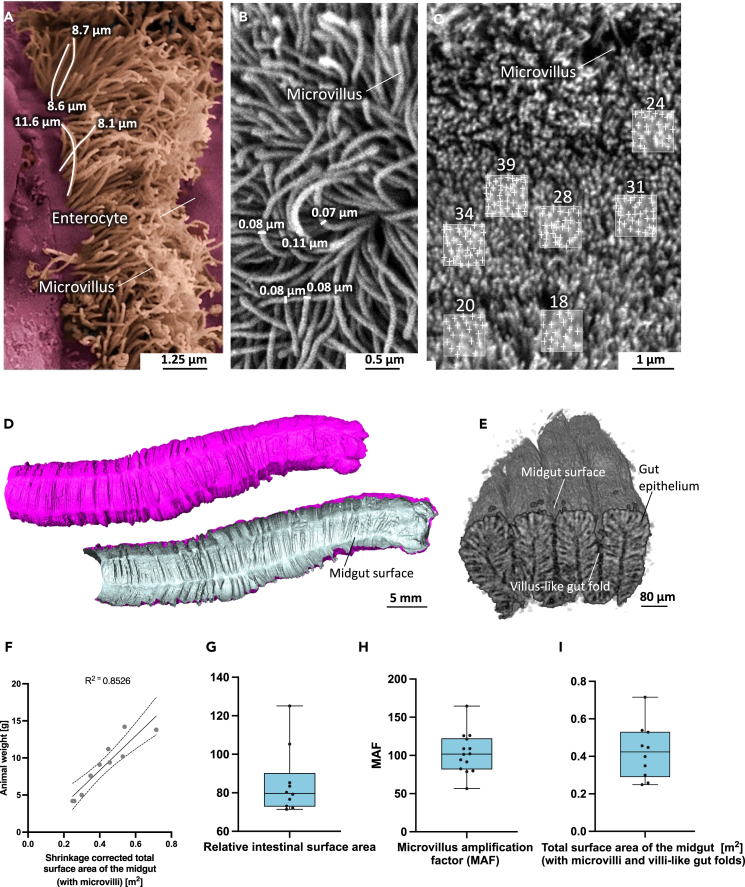
Quantification of the total surface area of the *M*. *sexta* midgut (A–C) Quantification of the mean length (A), diameter (B) and density (C) of microvilli by SEM. (D) Quantification of the midgut surface area by μCT. (E) Quantification of the surface area of deep villus-like midgut folds by nanoCT. (F) The midgut surface area is dependent on the specimen weight. The upper and lower dashed lines represent the 95% confidence interval. (G–I) Boxplots of the (G) relative intestinal surface area (RISA), (H) microvillus amplification factor (MAF), and (I) total surface area of the *M. sexta* midgut. Boxplots show the 25^th^ to 75^th^ percentiles, with whiskers extending to the minimum and maximum data values while including all data points. The center denotes the mean, and the center line signifies the median. For a detailed list of all findings, please refer to Table 1.

We used μCT to determine the mean shrinkage-corrected surface area of the midgut (without deep, villus-like midgut folds and microvilli) resulting in a value of 0.0013 ± 0.0004542 m^2^ (n = 10) (Table 1, Figures 7 and S6). We then repeated the measurement, combining μCT and nanoCT, resulting in a value of 0.00401 ± 0.001275 m^2^ (n = 10). The shrinkage-corrected length of the midgut determined by μCT was 46.91 ± 7.421 mm (n = 10). The microvilli density determined by SEM was (31.80 ± 3.846)/μm^2^ (n = 3). The mean microvillus diameter determined by SEM was 0.116 ± 0.02242 μm (n = 3), and the mean microvillus length determined by SEM was 9.036 ± 1.382 μm (n = 3). This equates to a mean microvillus surface area of 3.278 ± 0.821 μm^2^ (n = 3), a microvillus amplification factor (MAF) of 103.0 ± 26.57 (n = 3), and a relative intestinal surface area (RISA) of 85.17 ± 17.12 (n = 10) (Table 1, Figures 7 and S6). The estimated total surface area of the midgut was therefore 0.4242 ± 0.1455 m^2^ (n = 10) (Table 1) with mean surface area of 885 ± 184.5 cm^2^ per cm midgut length (n = 10) (Table 1).

**Table 1:** Calculated features of the midgut surface area, including the total surface area of the midgut

Measured feature in *M. sexta*	Mean	Standard deviation	n	Literature (Mouse^a^)	Mouse vs. *Manduca* comparison	*Manduca* vs. Mouse comparison
Shrinkage corrected surface of the midgut [m^2^] (no microvilli, no villi-like gut folds)	0.0013	±0.0004542	n = 10	/		
Surface of the midgut [m^2^] (no microvilli but with villi-like gut folds)	0.00401	±0.001275	n = 10	0.04	≈10x	
Shrinkage corrected length of the midgut [mm]	46.91	±7.421	n = 10	555	≈12x	
Microvilli density [#/μm^2^]	31.80	±3.846	n = 3	80.8	≈2.5x	
Microvillus diameter [μm]	0.116	±0.02242	n = 3	0.10		
Microvillus length [μm]	9.036	±1.382	n = 3	0.97		≈9x
Microvillus surface area [μm^2^]	3.278	±0.821	n = 3	0.32		≈10x
Microvillus amplification factor	103.0	±26.57	n = 3	26.2		≈4x
Relative intestinal surface area	85.17	±17.12	n = 10	109		
Total surface area of the midgut [m^2^] (with microvilli and villi-like gut folds)	0.4242	±0.1455	n = 10	1.41	≈3x	
Mean surface [cm^2^ ]/gut length [cm]	885.2	±184.5	n = 10	254.05		≈3x

a Casteleyn, C., Rekecki, A., Van der Aa, A., Simoens, P., and Van den Broeck, W. (2010). Surface area assessment of the murine intestinal tract as a prerequisite for oral dose translation from mouse to man. Lab. Anim *44*, 176–183. 10.1258/la.2009.009112.

### Hindgut

**Figure 8:**
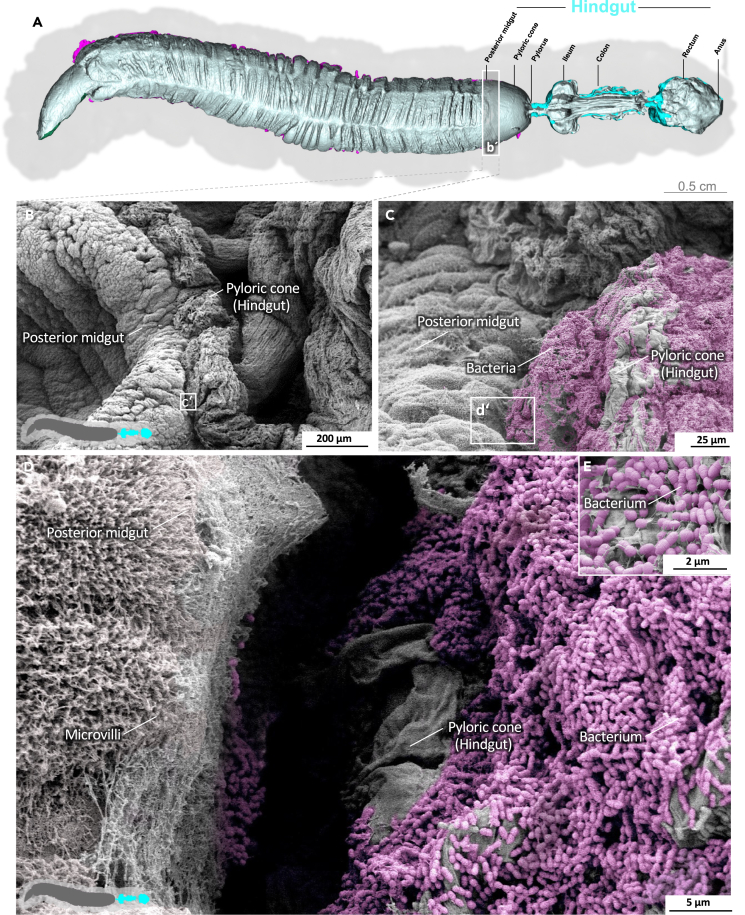
Hindgut (pyloric cone) of *M. sexta* In sharp contrast to the midgut, the folded intima of the pyloric cone (hindgut) is densely covered with a bacterial biofilm. A micro-tomographic surface overview of the digestive system of *M. sexta* (A) shows the localization (B′) of the SEM insets (B–D). The images in panels (C–E) are artificially colored to highlight the bacteria. A larger version of the image in panel (E) is provided as Figure S7.

**Figure 9:**
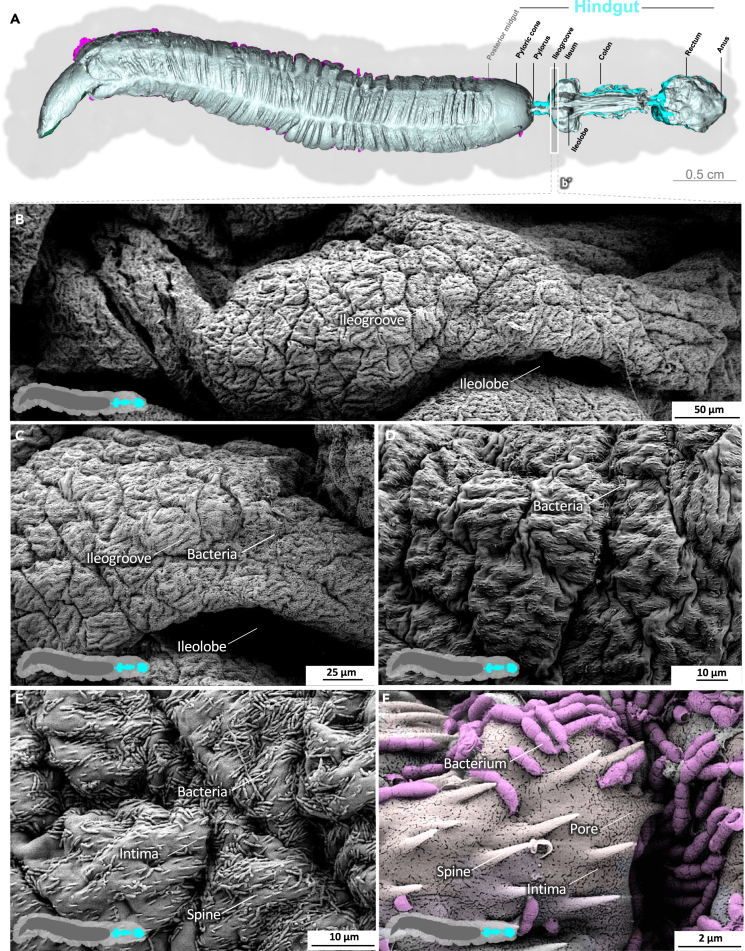
Hindgut (ileum) of *M. sexta* The folded intima is spiculated and densely populated with bacteria. At higher magnification, pores in the intima become evident. A micro-tomographic surface overview of the digestive system of *M. sexta* (A) shows the localization (B′) of the SEM insets (B–F). The image in panel (F) is artificially colored to highlight the bacteria.

**Figure 10:**
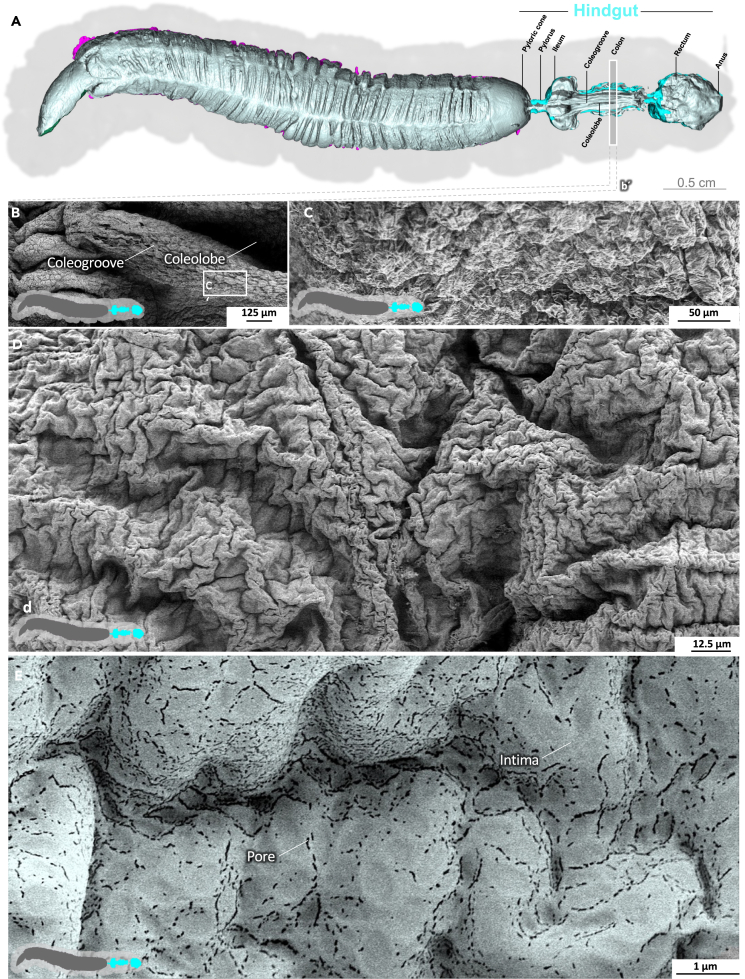
Hindgut (colon) of *M. sexta* The heavily folded intima of the colon lacks spines and bacteria. The intima has spherical imprints (sacculations) at higher magnification and pores are visible. A micro-tomographic surface overview of the digestive system of *M. sexta* (A) shows the localization (b′) of the SEM insets (B–E). The image in panel (E) is artificially colored to highlight the pores.

**Figure 11:**
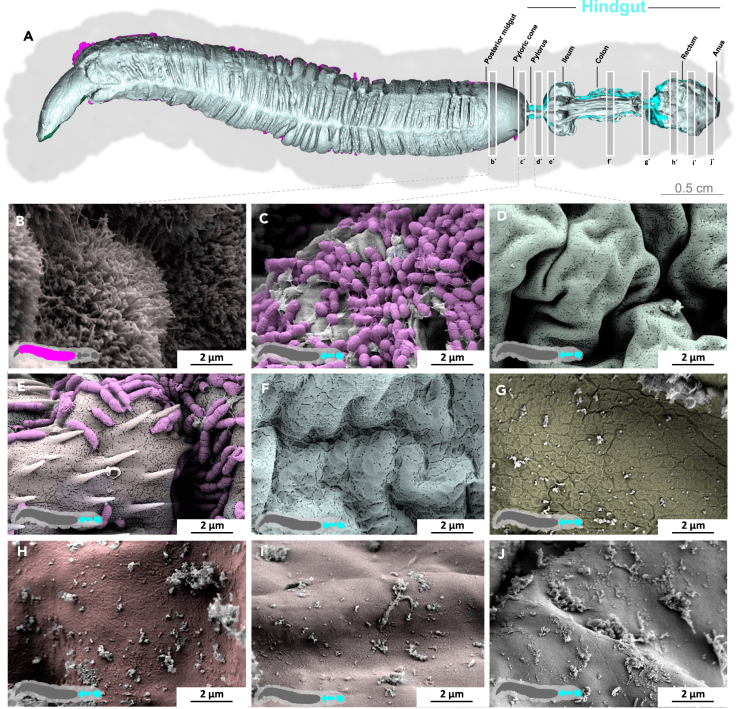
Hindgut (overview) of *M. sexta* The diverse surface structure of the *M. sexta* hindgut is shown at 10,000× magnification. In contrast to the more anterior hindgut parts, the rectum has a smooth intima. A micro-tomographic surface overview of the digestive system of *M. sexta* (A) shows the localization (B′) of the SEM insets (B–J). The images in panels (B–I) are artificially colored to highlight diverse surface features.

The transition from the posterior midgut to the first part of the hindgut, the pyloric cone, is marked by an abrupt change in surface structure (Figure 8; Video S7). The cuticle-coated intima of the pyloric cone abruptly displaces the enterocytes mounted with microvilli. Unlike the peritrophic matrix, the pyloric cone is densely populated with enterococci. Next, the pylorus shows the characteristic armature of spiculated pads at the pyloric valve (Figure S8).^32^^,^^33^ The dense population of bacteria persists through the ileum and the cuticle surface features numerous spikes (Figures 9, S9, and Video S8). Densely spiculated fields alternate with spines arranged in lines (Figure S9). In general, the direction of the lines is not uniform, and in some cases, they form perpendicular arrays, suggesting a role in shredding the passing peritrophic matrix (Figure S9). At higher magnification, pores in the intima become evident (Figure 9; Video S8). The colon is heavily folded and devoid of spines and bacteria (Figure 10; Video S9), but the intima still features pores. The intima at the colon–rectum transition shows spherical imprints or sacculations (Figure S10). Also, significant amounts of debris and remains of the peritrophic matrix are present in this area. Finally, the rectum shows a smooth and lightly folded intima without spines or pores but with significant amounts of debris (Figures 11H–11J, S11, and Video S10). We previously determined the volume of the L5d6 hindgut (without the pyloric cone), reporting a value of 0.208 ml with a mean area of 348.2 mm^2^.^13^

## Discussion

We have developed a comprehensive gut surface atlas of the model insect *Manduca sexta*, which documents the physiological standard of healthy animals as a reference for the qualitative phenotyping of gut-associated pathologies such as gut inflammation.^16^ The atlas also allowed us to estimate the resorptive surface area of the midgut, which will facilitate comprehensive allometric scaling for enteral dose conversion from insects to mammals.

### Foregut

The foregut has the primary functions of pre-digestion, food storage (as a crop), supplying food to the midgut. The foregut also facilitates regurgitation, an essential defensive strategy against predators.^34^^,^^35^ The intima of the foregut is folded and has no spines, as reported in other lepidopteran species.^36^^,^^37^^,^^38^^,^^39^^,^^40^^,^^41^ In line with its function, the cuticle of the *M. sexta* foregut has a very low permeability compared to the cuticle of the ileum and rectum.^42^

### Midgut

The midgut is the principal site for digestion and nutrient resorption.^13^^,^^29^^,^^34^ The primary cell types found in the *M. sexta* midgut epithelium (and that of other lepidopterans) are columnar cells and goblet cells. The columnar cells are responsible for the secretion of digestive enzymes and the absorption of nutrients, whereas goblet cells actively pump K^+^ from the hemolymph into the gut lumen using a K^+^/2H^+^ antiporter mechanism, thereby maintaining an alkaline environment in the midgut as is typical for most lepidopterans.^13^^,^^34^^,^^43^

Columnar cells and goblet cells undergo significant morphological changes along the anterior–posterior axis.^13^^,^^29^^,^^34^ In the anterior midgut, columnar cells have club-shaped microvilli on their apical surfaces, and cytoplasmic bridges connect them to form a complex meshwork. Regular microvilli are found only from the rear of the anterior midgut toward the middle midgut.^13^^,^^29^^,^^34^ In the anterior midgut, goblet cells feature a widened cavity located at the cell base with a long neck connecting it to the lumen. Microvilli lining this cavity contain mitochondria, and the nucleus is at the level of the widened cavity.^29^ In contrast, goblet cells in the posterior midgut feature a cavity restricted to the upper part of the cell above the nucleus, giving them a goblet shape. Microvilli in this region lack mitochondria.^29^ Both types of goblet cell have a valve of closely spaced microvilli at the cavity entrance.^44^ These differences are not graded but change abruptly between midgut regions in *M. sexta*.^13^^,^^29^^,^^34^ Such physiological variations should not be confused with the symptoms of gut inflammation and infection.^16^ Because goblet cell microvilli (projections of the goblet cell cave membrane) are not part of the resorptive surface, we did not include them in the quantification of the midgut surface area.

Extensive epithelial erosion, a hallmark of conditions such as gut inflammation and infectious colitis in both mammals and insects, is easy to recognize by SEM.^16^^,^^45^ Changes in plasma membrane dynamics and enterocyte morphology are typical of these conditions, including enterocyte blebbing,^46^^,^^47^ shedding^23^^,^^46^ and purging^20^ as well as the shedding or expulsion of microvilli.^20^^,^^21^^,^^47^ Previously, we and others documented these SEM-accessible phenotypes in *M. sexta* and observed enterocyte swelling, shedding and membrane extrusion^16^^,^^27^ as well as blebbing and necrosis^16^ augmented by the shortening and loss of microvilli.^16^^,^^26^^,^^27^

Data concerning the resorptive midgut surface are needed for allometric dose scaling from caterpillars to mice in preclinical studies. We therefore combined our SEM, μCT and nanoCT data to estimate the total resorptive midgut surface area of L5d6 *M. sexta* larvae, revealing an average value of 0.42 m^2^ (Table 1) although one particularly large specimen had a resorptive midgut surface area of 0.71 m^2^ (Figure 7F). The average midgut surface area value of *M. sexta* corresponds to approximately one-third of the surface area of the mouse intestine.^30^ This is impressive because the mouse intestine is more than 10 times longer. We also found that *M. sexta* lacks intestinal villi and has a lower density of microvilli compared to mammals.^48^ However, this is partially compensated by the presence of microvilli that are nine times longer than those in mice.^29^ Accordingly, the MAF of *M. sexta* is four times higher than that of mice. Instead of villi, deep villus-like midgut folds are present in *M. sexta*. This enhances and refines our previous studies, which showed comparable intestinal volumes in mice and late-instar *M. sexta* larvae.^13^ Even so, interspecies allometric scaling from laboratory animals to humans for dose conversion is one of the most contentious areas in clinical pharmacology and should be evaluated carefully, even when comparing different mammals.^49^^,^^50^^,^^51^ Nevertheless, our data will help to improve the assessment of allometric scaling.

The peritrophic membrane is another central feature of the midgut,^13^^,^^52^ consisting of a mucinous intestinal lining reinforced with chitin.^3^^,^^53^ The *M. sexta* peritrophic matrix consists of 60% protein and 40% chitin.^54^ Insect intestinal mucin (IIM) and chitin-binding proteins connect the chitin fibrils and form a gel-like membranous structure.^53^ By convention, two different types of peritrophic matrices are recognized in insects.^52^ Type 1 matrices are secreted from extensive areas of the midgut epithelium and consist of multiple laminae,^52^ whereas type 2 matrices are produced by specialized cells of the stomodeal and mesenteric epithelia and consist of only a single layer.^52^ By the same convention, *M. sexta* has a type 1 peritrophic matrix.^52^^,^^55^^,^^56^ Interestingly, IIMs that have been described thus far share many similarities with MUC2, one of the core components of mucus in the human intestine and a key player in the pathogenesis of ulcerative colitis.^53^^,^^57^^,^^58^ The primary function of intestinal mucus in both humans and insects is to protect the underlying epithelium from bacteria.^3^^,^^58^^,^^59^^,^^60^

We found that the endoperitrophic surface of the peritrophic matrix in *M. sexta*, especially the pyloric cone and ileum, are populated by enterococci. Previously, we characterized the *M. sexta* gut microbiome and found the community was dominated by two species of enterococci, both acting as protective symbionts.^16^ Given their location and morphology,^31^ the bacteria detected on the peritrophic matrix appear to be the same species we previously characterized. Importantly, these bacteria were present on the endoperitrophic sheet but not in the ectoperitrophic space in *M. sexta*, agreeing with other studies claiming that the peritrophic matrix is an effective bacterial barrier.^61^

The main structural difference between insect and mammalian intestinal mucins is the presence of chitin in the peritrophic matrices of insects.^53^ Human intestinal mucus forms a gel-like, shapeless lining within the intestine, with a typical thickness of 50–200 μm.^58^ In contrast, the peritrophic matrix in *M. sexta* and other insects is considerably thinner, typically 1–12 μm.^13^^,^^55^^,^^56^ Therefore, reinforcing this thinner mucin layer with chitin fibrils may help to achieve mechanical strength and stability equivalent to that observed in mammals.^53^

### Hindgut

Toxic nitrogenous waste from the Malpighian tubules is secreted into the lumen of the pyloric cone.^13^ The predominant waste product in *M. sexta* is uric acid.^62^ Some enterococci, such as *Enterococcus faecalis*, can metabolize uric acid.^63^^,^^64^ It therefore seems plausible that these bacteria have densely populated the pyloric cone and the entire hindgut downstream to the pylorus. On closer inspection, we found pores in the areas of the hindgut colonized by bacteria. To our knowledge, such pores have not been described in other lepidopteran species and their function is unclear. The main site of water reabsorption in *M. sexta* is the water-permeable intima of the rectum,^65^ but we did not observe any pores there, making a potential role in the augmentation of water reabsorption unlikely. Because the pore-lined region, apart from the colon, is densely colonized with bacteria, the pores may help the underlying epithelium to control the bacterial population and facilitate an immune response if necessary. This hypothesis is supported by the high DUOX expression level in the larval hindgut and the secretion of prophenoloxidase.^66^^,^^67^ We also observed spines in the hindgut intima organized in pads, lines, and fields, which may help to grind the incoming peritrophic matrix, exposing the bacteria from the endoperitrophic space to the hindgut immunosurveillance.

### Summary

Our comprehensive SEM-based atlas provides a detailed overview of the ultrastructure of the gut surface in *M. sexta*. Given its role as a model of gut inflammation and host–microbe interactions, we have developed a reference standard of healthy *M. sexta* individuals, identifying distinct morphological changes across the anterior, middle and posterior midgut. This provides a valuable tool for the SEM-based screening of abnormal gut-related traits such as inflammation and infection-induced colitis. Additionally, we estimated the total resorptive midgut surface area in L5d6 *M. sexta* larvae. The surface area of 0.42 m^2^ highlights the remarkable size of the gut despite the absence of villi, relying instead on deep villus-like folds housing exceptionally long microvilli. These data are necessary for allometric scaling and accurate dose conversion from *M. sexta* to mammals in preclinical studies, while supporting the 3R principles. We also revealed the unique colonization pattern of enterococci, forming dense biofilms in the pyloric cone and downstream of the ileum, accompanied by distinctive pore and spine structures within the hindgut cuticle. These findings suggest the pores and spines may be involved in the immunosurveillance of the underlying gut epithelium.

### Limitations of the study

This study reports a reference surface atlas of *M. sexta* larvae in the fifth larval stage on development day 6 (L5d6). Therefore, the reader should draw conclusions for developmental stages not represented in this study with caution. Furthermore, the impact of the animals’ sex on the intestinal ultrastructure could not be ascertained due to the uncertainty of sex determination in *M. sexta* larvae. In addition, SEM analysis can be prone to shrinkage artifacts. Therefore, additional care is needed in the evaluation of surface details at high magnifications (at and beyond 10,000×).

## STAR★Methods

### Key resources table

**Table:** 

REAGENT or RESOURCE	SOURCE	IDENTIFIER
**Chemicals, peptides, and recombinant proteins**		

Iodixanol (Visipaque)	GE Healthcare	Cat# 1133992
Ethyl acetate	Bioform	Cat# A44a
Diatrizoate (Gastrografin)	Bayer	H/28/2842
Conductive Silver LS200N	Plano	# G3303B
Glutaraldehyde for electron microscopy	Carl Roth	# 4995.1

**Experimental models: Organisms/strains**		

*Manduca sexta* (Lepidoptera; Sphingidae), (Linnaeus, 1763) L5d6	Universität Giessen, Germany	Dr. Anton Windfelder

**Software and algorithms**		

NRecon v1.7.3.0	Bruker	https://www.bruker.com
DataViewer v16.0.0	Bruker	https://www.bruker.com
CTVOX v3.3.1	Bruker	https://www.bruker.com
CTAn v1.20.8.0	Bruker	https://www.bruker.com
Amira 3D 2022.1	Thermo Fisher Scientific	https://www.thermofisher.com
Final Cut Pro 10.6.10	Apple	https://www.apple.com
GraphPad Prism 9.5.0	Insight Partners	https://www.graphpad.com
Adobe Photoshop 2020 21.2.2	Adobe	https://www.adobe.com

### Resource availability

#### Lead contact

Information on resources and reagents should be directed to the lead contact, Prof. Dr. Andreas Vilcinskas (Andreas.vilcinskas@agrar.uni-giessen.de).

#### Materials availability

Any additional information required to reanalyze the data reported in this paper is available from the lead contact upon request (Andreas.vilcinskas@agrar.uni-giessen.de).

#### Data and code availability

• Data is available from the lead contact Prof. Dr. Andreas Vilcinskas (Andreas.vilcinskas@agrar.uni-giessen.de).
• This study did not create code.
• Any additional information required to reanalyze the data reported in this work is available from the lead contact upon reasonable request.

### Experimental model and subject details

*Manduca sexta* (Lepidoptera; Sphingidae) was reared from the egg stage at the University of Giessen, Germany. In brief, the imagos were kept in flight cages with tobacco plants. Eggs were collected two times a week from the plants. The larvae were then maintained on an artificial diet^16^ in a controlled insect incubator set at 24 °C and 40 % relative humidity while exposed to long-day conditions with a 17 h photoperiod. It is problematic to determine the sex of *M. sexta* larvae, so we did not record the sex of our specimens. Insects fall outside the scope of regulations covering animal experiments in Europe and the USA, so no ethical approval was required.

### Method details

#### Scanning electron microscopy (SEM)

L5d6 larvae were sacrificed with ethyl acetate (Bioform, Nürnberg, Germany) in a killing jar (n = 9). The intestinal tract was opened dorsally, and the peritrophic matrix was removed or left in the intestine, depending on what was to be examined. The gastrointestinal systems (with or without Peritrophic matrix) were dissected in phosphate-buffered saline (PBS) and fixed in 2 % paraformaldehyde (PFA) and 0.5 % glutaraldehyde in PBS at room temperature for 3 h. The fixative solution was then diluted 1:10, and the samples were stored at 4 °C. Midgut samples were rinsed first in PBS and then ultrapure water, followed by dehydration through an ethanol series on ice (30 %, 50 %, 70 %, 80 %, 90 %, 96 %, 99.8 %, and 100 %, 10 min each). After critical point drying using a CPD 030 device, the samples were sputter-coated with gold using an SCD 004 instrument (all equipment was obtained from Balzers, Balzers, Liechtenstein) and mounted on aluminum stubs using Conductive Silver LS200N (Plano, Wetzlar, Germany). The samples were then analyzed using an EM9DSM982 scanning electron microscope (Zeiss, Oberkochen, Germany). For clarity, some SEM images were colored in Adobe Photoshop 2020 21.2.2 (Adobe Systems, San Jose, CA, USA). Videos S1, S2, S3, S4, S5, S6, S7, S8, S9, and S10 were generated in Amira Animation Director 2022.1. (Thermo Fisher Scientific, Waltham, MA, USA) and annotated using Final Cut Pro 10.6.10 (Apple, Cupertino, CA, USA).

#### Micro-computed tomography (μCT)

The μCT procedure is described in detail elsewhere.^13^ Briefly, the diet used to rear *M. sexta* larvae was submerged in iodixanol (Visipaque 320; GE Healthcare, Solingen, Germany) and L5d6 larvae were fed *ad libitum* for 12 h before euthanization with ethyl acetate (Biofrom, Nürnberg, Germany) in a killing jar. The animals were fixed in 4 % PFA before scanning, and the scans were corrected for shrinkage as previously described.^13^ The specimens were examined in a high-energy SkyScan 1173 μCT device (Bruker, Kontich, Belgium) with the following parameters: source voltage 50 kV, source current 160 μA, image pixel size 19.6 μm, rotation step 0.3 °, and frame averaging of 2. Image reconstruction, preprocessing and segmentation described elsewhere.^13^^,^^68^

#### Nano-computed tomography (NanoCT)

The deep villus-like midgut folds were analyzed using a Skyscan 2011 NanoCT device (Bruker, Kontich, Belgium). Dissected midgut tissue was immersed in 1 % iodine in 70 % ethanol for 5 days, followed by critical point drying as described above (Figure 7E). The samples were scanned using Basotect foam^69^ and examined with the following parameters: source voltage 35 kV, source current 190 μA, image pixel size 1.52 μm, rotation step 0.2 °, and frame averaging of 4. Image reconstruction, preprocessing and segmentation are described elsewhere.^13^

### Quantification and statistical analysis

The midgut surface area (without the pyloric cone) was determined as previously described^13^ using Amira 3D 2022.1 (Thermo Fisher Scientific, Waltham, MA, USA). Briefly, the orally applied contrast agent (iodixanol) allowed the detailed surface analysis of the midgut region (n = 10). We found μCT quantification suitable for accessing the overall surface but the pixel size of 19.6 μm gave insufficient resolution to access the deep hidden surfaces of villus-like inner midgut folds (<10 μm). We, therefore, enhanced the μCT data with nanoCT data at a resolution of 1.52 μm (Figure 7E). The midgut length was measured using the iodixanol midgut surface models in Amira 3D. The body surface area (BSA) was also determined in Amira 3D using μCT scans. Microvillus density, length (from tip to base, l), and diameter (2r) were determined using representative SEM images (Figures 7A–7C). The microvillus surface area was calculated using the equation A=2rπl as previously described.^30^ To evaluate the augmentation of the intestinal surface area by microvilli (the MAF), we multiplied the density of microvilli (#/mm^2^; Figure 7C) by the microvillus surface area (mm^2^). To estimate the total surface area (with microvilli) for each specimen, the mean microvillus surface area was multiplied by the mean density of microvilli (#/qm^2^). The shrinkage-corrected total surface area of the midgut was obtained by multiplying these values with the surface area of the midgut determined by μCT (n = 10). The RISA was determined as previously described^30^^,^^48^ by dividing the intestinal surface area by the BSA. Simple linear regression was used to show the relationship between specimen weight and the total surface area of the midgut. Descriptive statistics and regression were visualized and calculated using GraphPad Prism 9.5.0 (Insight Partners, New York, NY, USA). Data in bar charts are presented as means ± standard deviations. Scatterplots show 95% confidence intervals as dashed lines and include a trend line. Boxplots display the 25^th^ to 75^th^ percentiles, with whiskers extending to the minimum and maximum data values while including all data points. The center denotes the mean, and the center line signifies the median.
