# Supplementary material for: An enteric ultrastructural surface atlas of the model insect Manducasexta
Source: iScience. 2024 Mar 4;27(4):109410. doi: 10.1016/j.isci.2024.109410 (PMC10981077; doi:10.1016/j.isci.2024.109410)

iScience, Volume *27*

Supplemental information

An enteric ultrastructural surface atlas of the model insect *Manduca sexta*

Anton G. Windfelder, Jessica Steinbart, Leonie Graser, Jan Scherberich, Gabriele A. Krombach, and Andreas Vilcinskas

Figure S1: Foregut, related to figure S2. A folded intima with a rough surface dominates the cuticle-lined surface of the foregut. A micro-tomographic surface overview of the digestive system of *M. sexta* (**a**) shows the localization (**b'**) of the SEM insets (**b**-**f**). The image in panel (**f**) is artificially colored to highlight the intima.

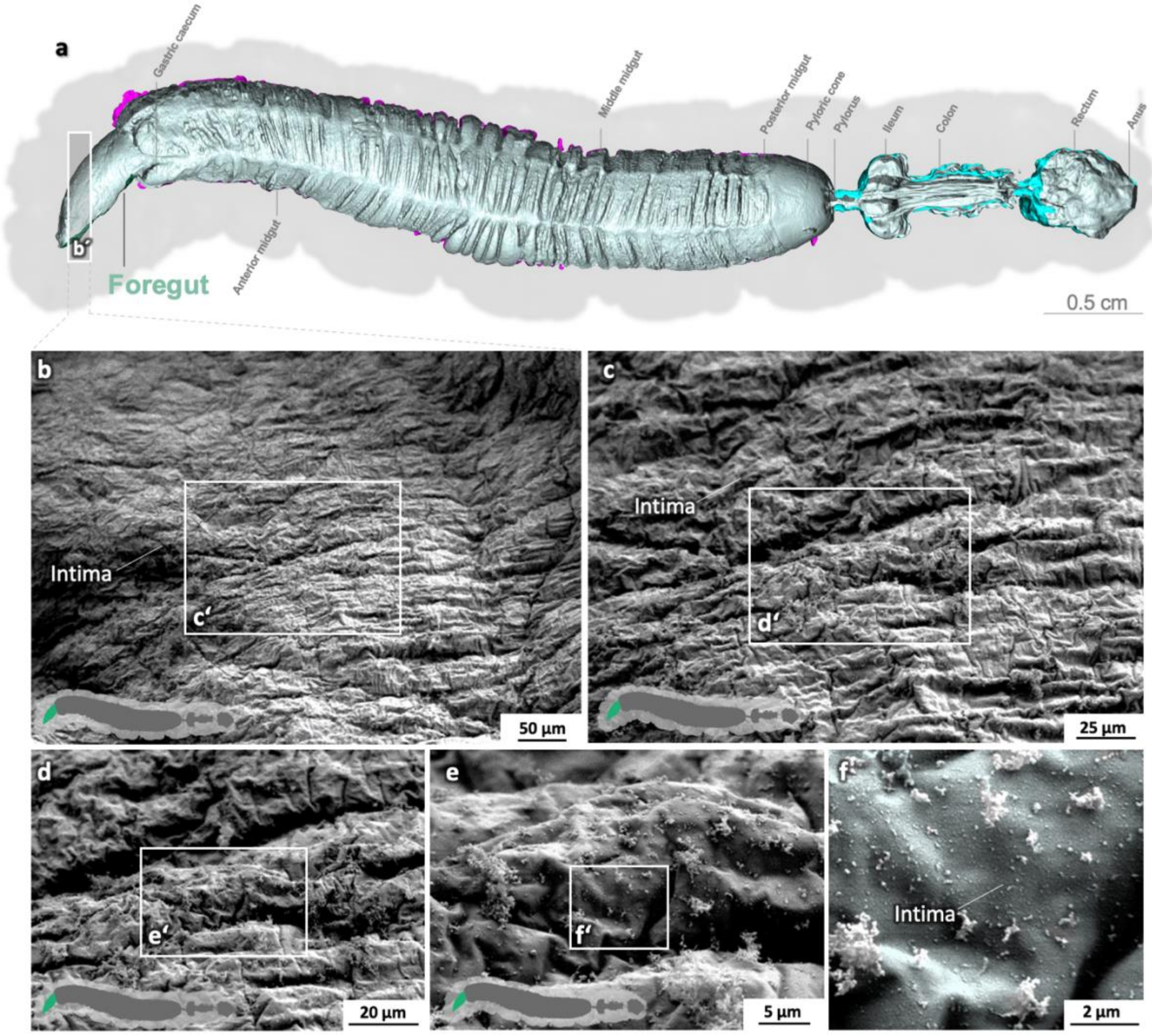

Figure S2: Foregut, related to figure S1. A folded intima with a rough surface dominates the cuticle-lined surface of the foregut. A micro-tomographic surface overview of the digestive system of *M. sexta* (**a**) shows the localization of the SEM insets (**b**-**f**).

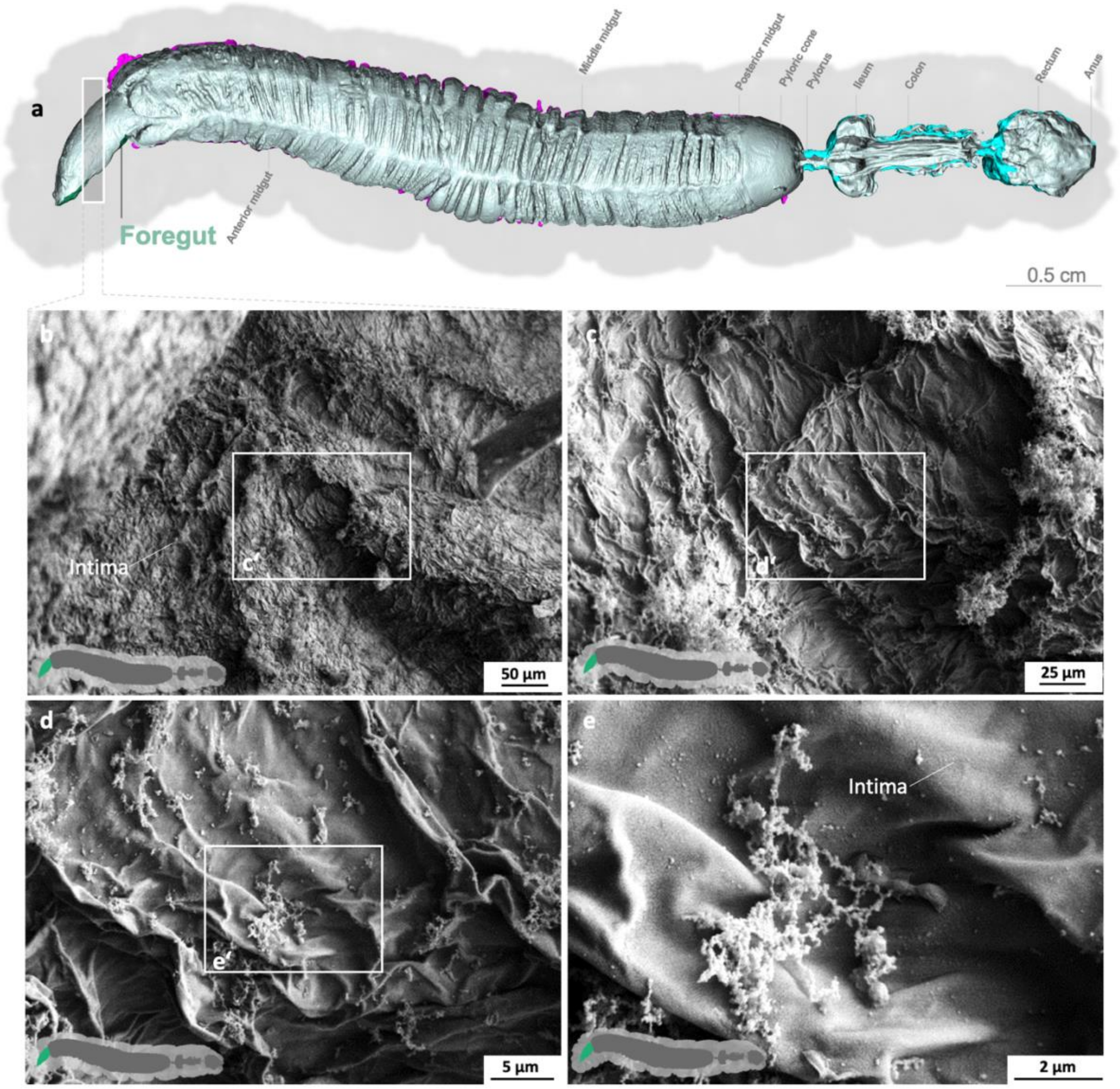

Figure S3: Foregut. A folded intima covers the stomodeal valve of *M. sexta*. A micro-tomographic surface overview of the digestive system of *M. sexta* (**a**) shows the localization of the SEM insets (**b-e**).

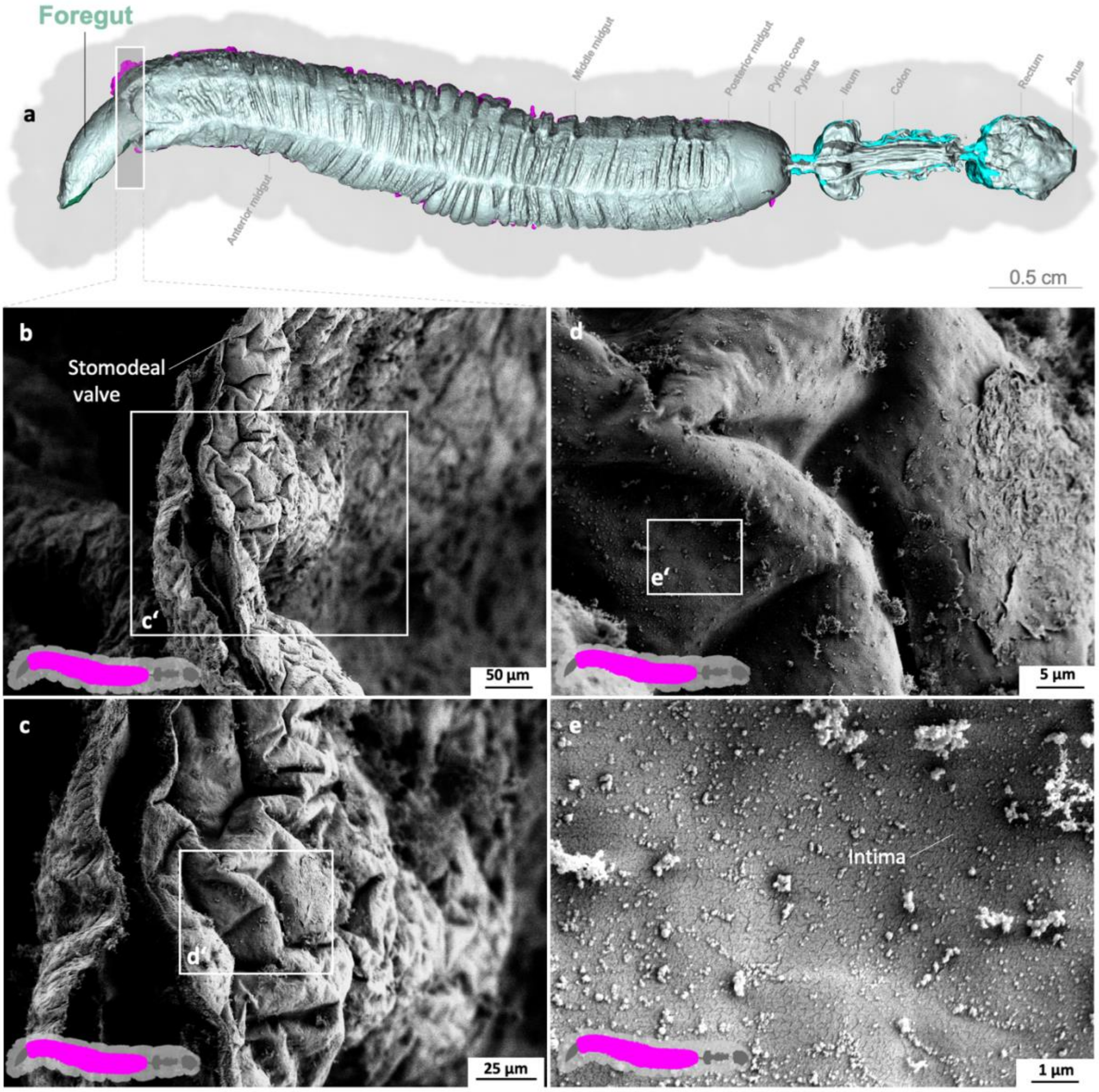

Figure S4: Gastric caecum. Elongated cylindrical structures characterize this area. A micro-tomographic surface overview of the digestive system of *M. sexta* (**a**) shows the localization of the SEM insets (**b-f**).

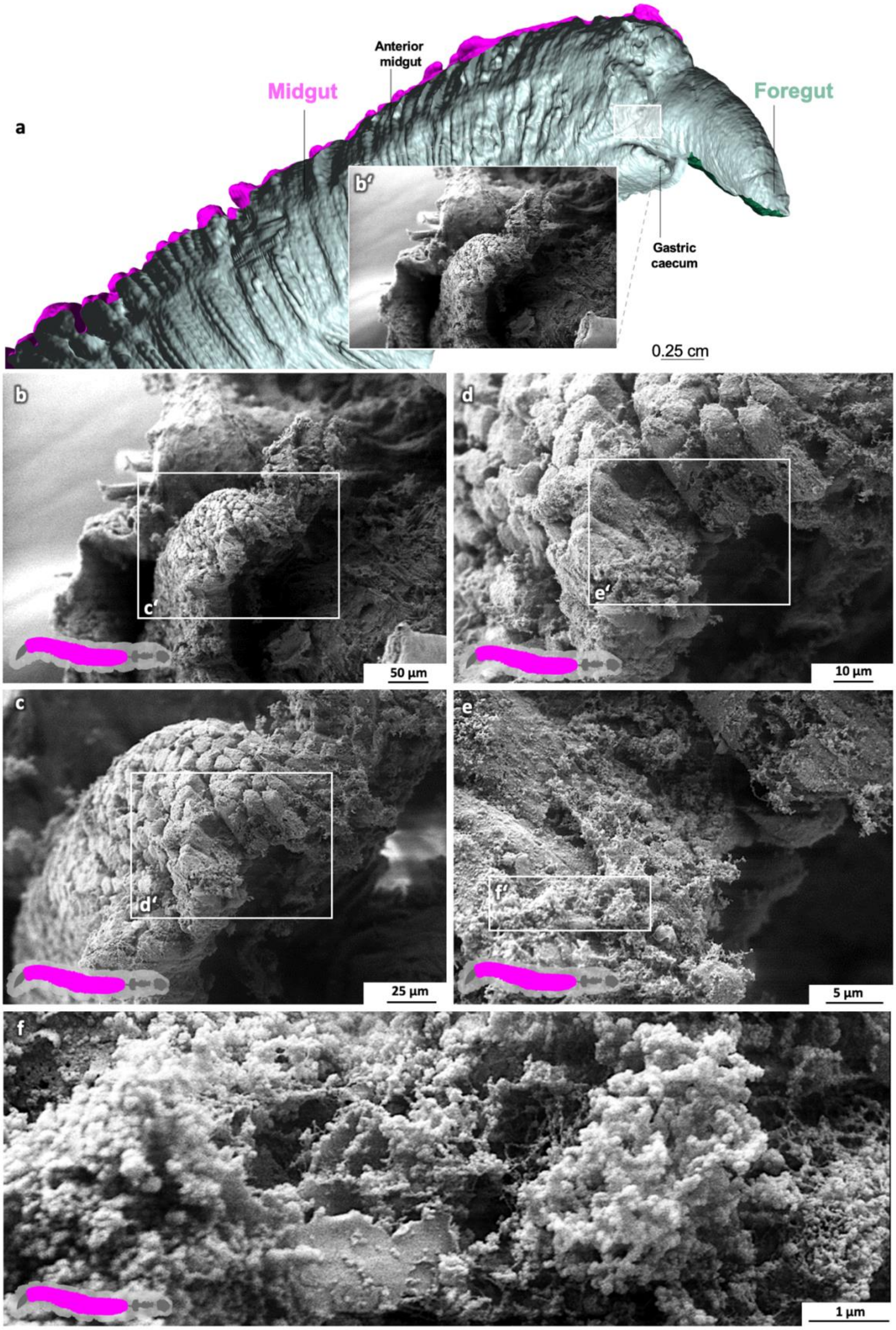

Figure S5: Middle midgut with a detached peritrophic matrix, related to figure 4. Only the remaining peritrophic matrix is shown. A micro-tomographic surface overview of the digestive system of *M. sexta* (**a**) shows the localization of the SEM insets (**b**, **c**).

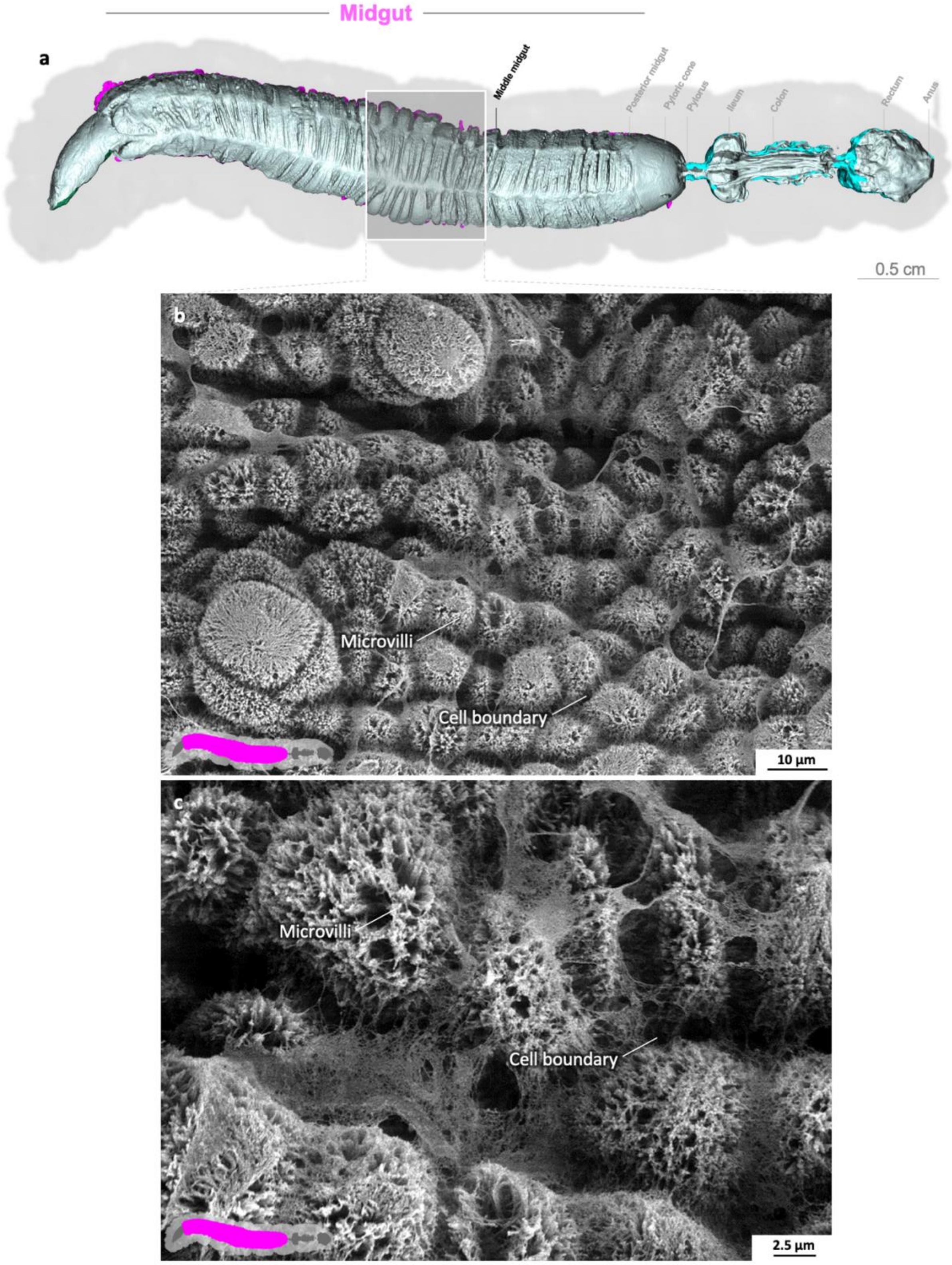

Figure S6: Quantification of the total surface area, related to figure 7 and table 1. Boxplots of (**a**) the shrinkage-corrected surface of the midgut (without villus-like midgut folds and microvilli), (**b**) the shrinkage- corrected surface of the midgut (with villus-like midgut folds and no microvilli), (**c**) the shrinkage-corrected length of the midgut, (**d**) density of microvilli, (**e**) microvillus diameter, (**f**) microvillus length, and (**g**) microvillus surface area. The data in the bar charts are means ± standard deviations. Scatterplots show 95% confidence intervals as dashed lines and include a trend line. Boxplots show the 25th to 75th percentiles, with whiskers extending to the minimum and maximum data values while including all data points. The center denotes the mean, and the center line signifies the median.

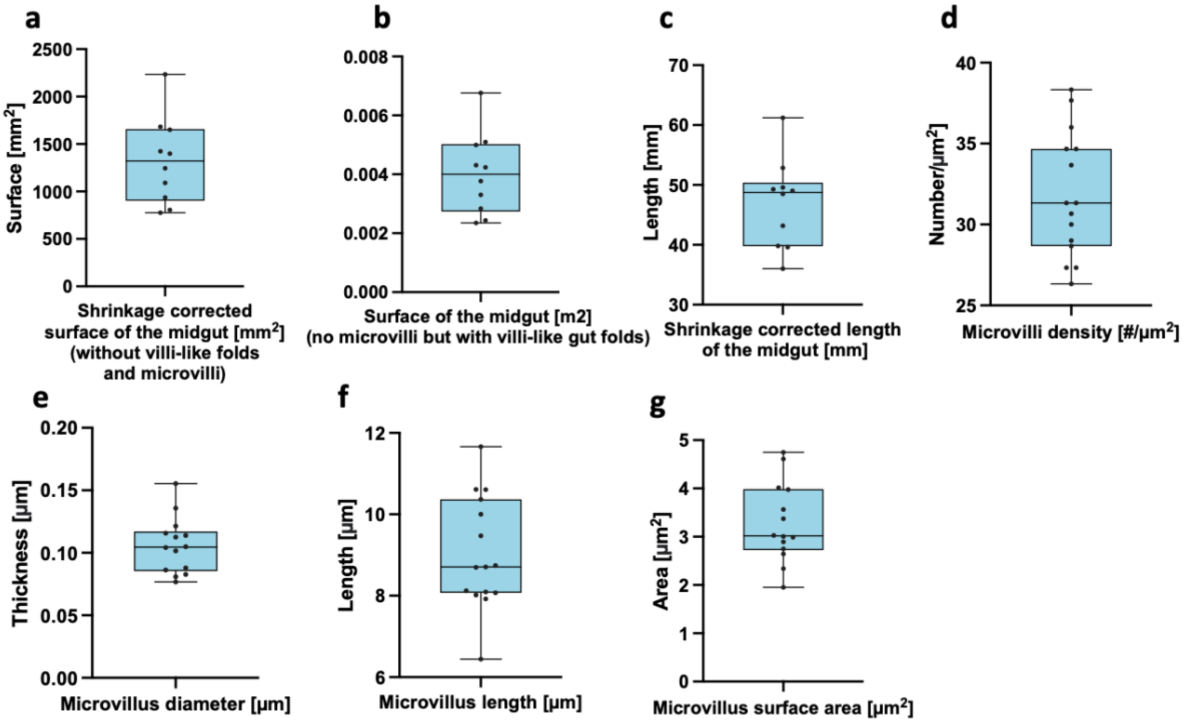

Figure S7: Hindgut (pyloric cone), related to figure 8. The folded intima of the pyloric cone is densely covered with a bacterial biofilm. A micro-tomographic surface overview of the digestive system of *M. sexta* (**a**) shows the localization of the SEM inset (**b**).

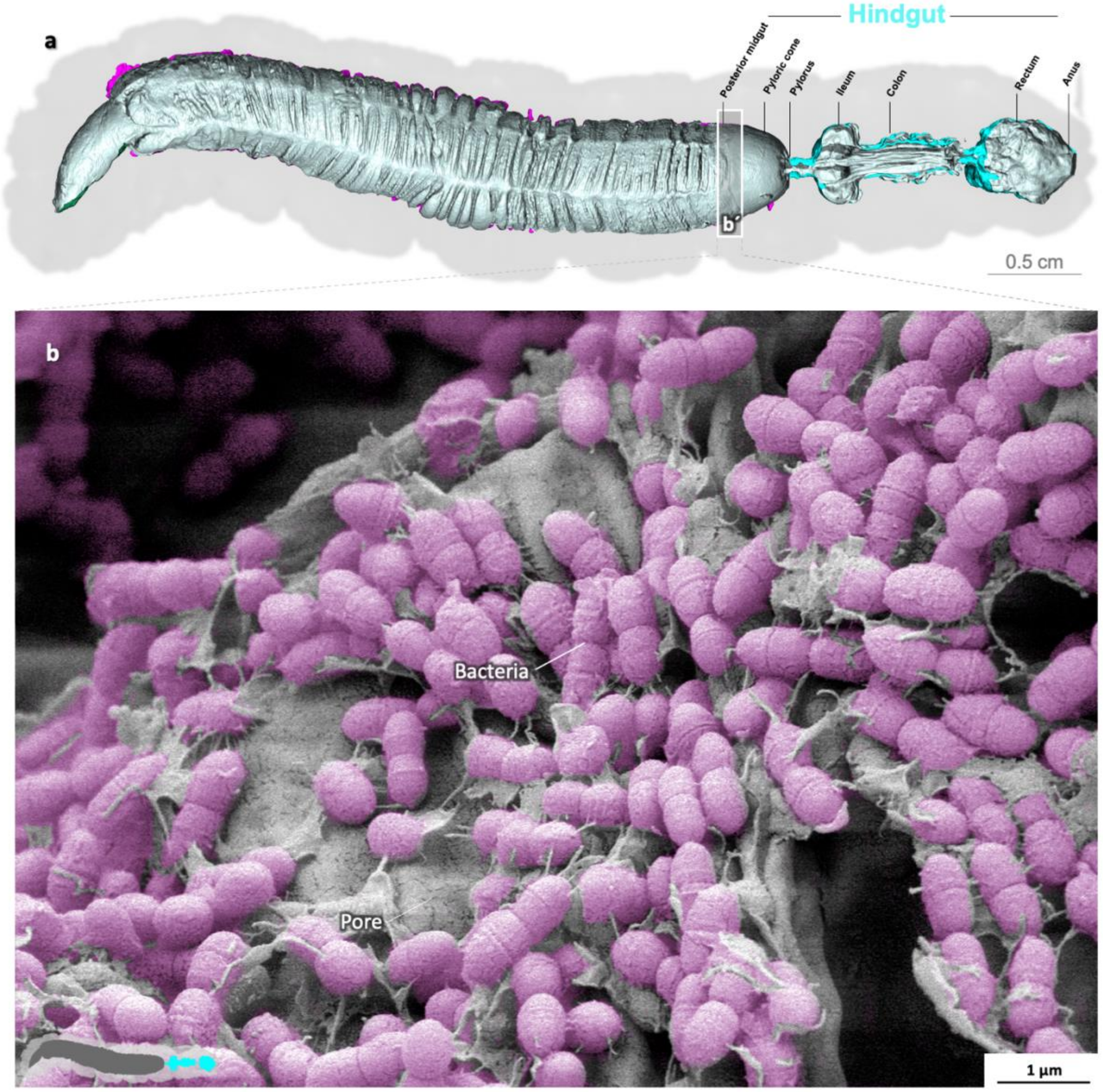

Figure S8: Hindgut (pyloric valve), related to figure 9. The intima at the pyloric valve shows an armature of spines. Three pads of spines are situated at the top of each pyloric groove. A micro-tomographic surface overview of the digestive system of *M. sexta* (**a**) shows the localization of the SEM insets (**b**-**e**).

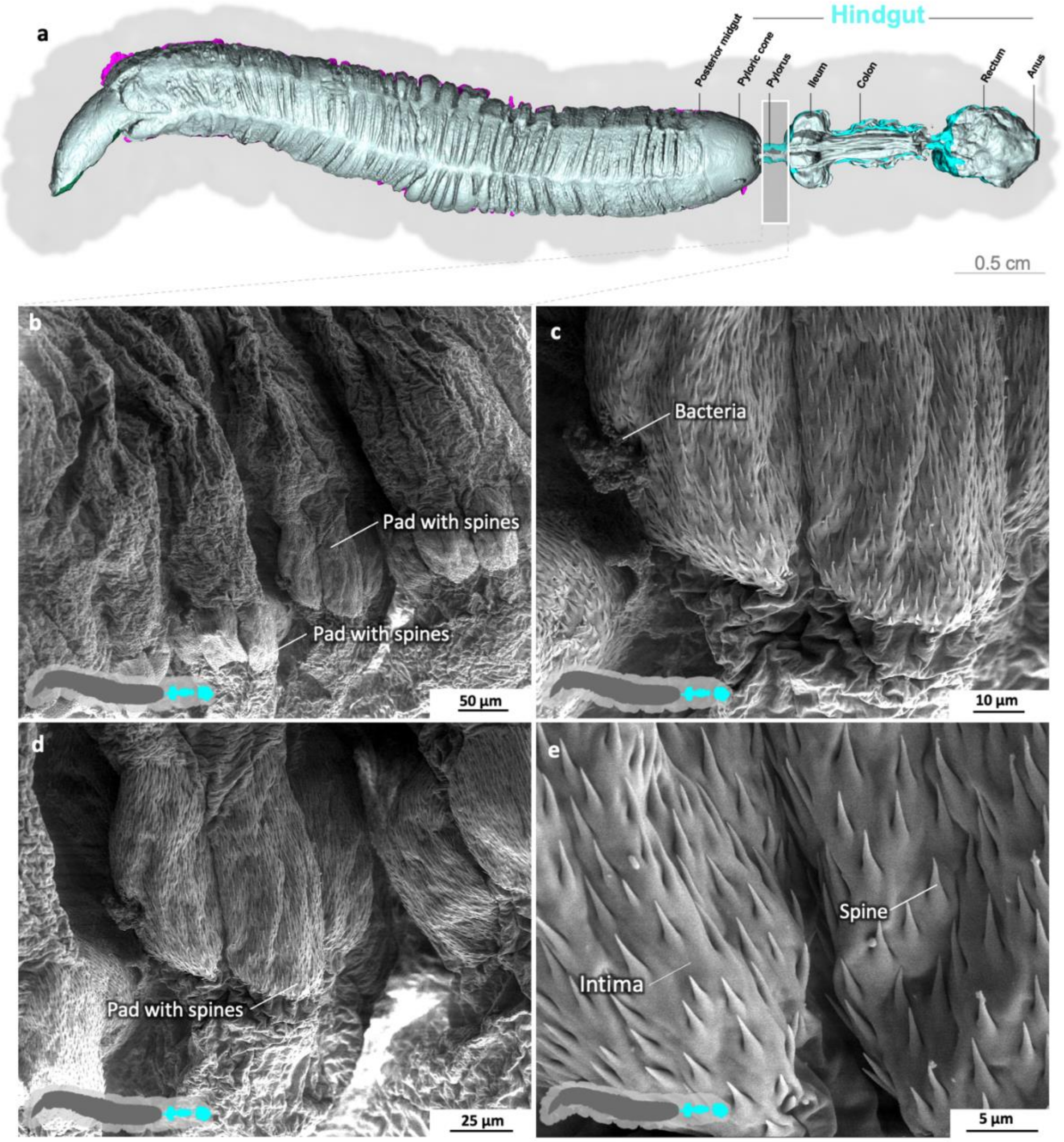

Figure S9: Hindgut (pyloric–ileum transition), related to Figure 9. The folded intima shows lines of spines that are partly arranged in an orthogonal array (arrows). Bacteria heavily colonize parts of the pyloric–ileum transition. A micro-tomographic surface overview of the digestive system of *M. sexta* (**a**) shows the localization of the SEM insets (**b**, **c**).

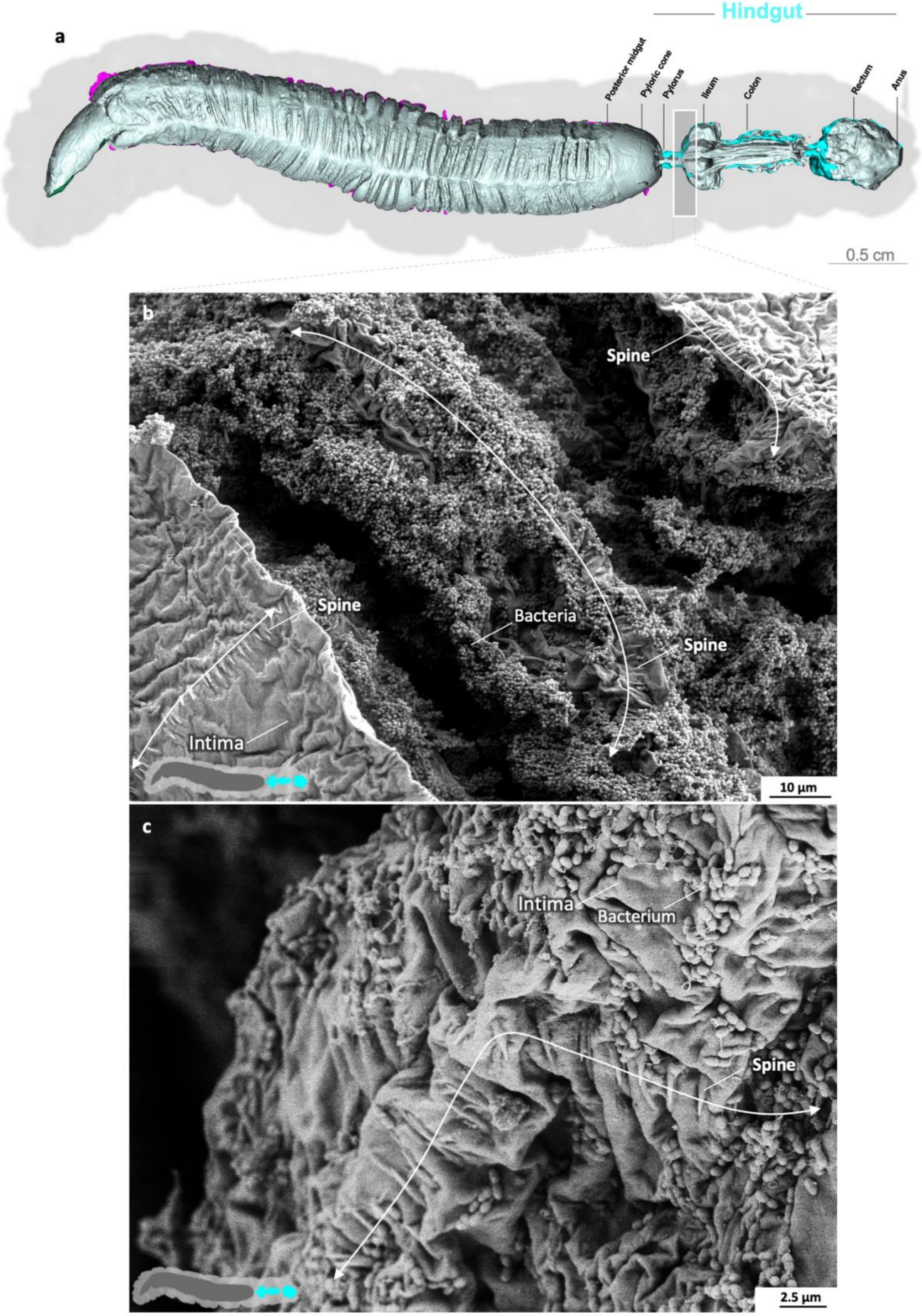

Figure S10: Hindgut (colon–rectum transition), related to figure 11. The intima at the colon–rectum transition shows spherical imprints or sacculations. A piece of the peritrophic matrix is visible at the top left side. A micro-tomographic surface overview of the digestive system of *M. sexta* (**a**) shows the localization of the SEM inset (**b**).

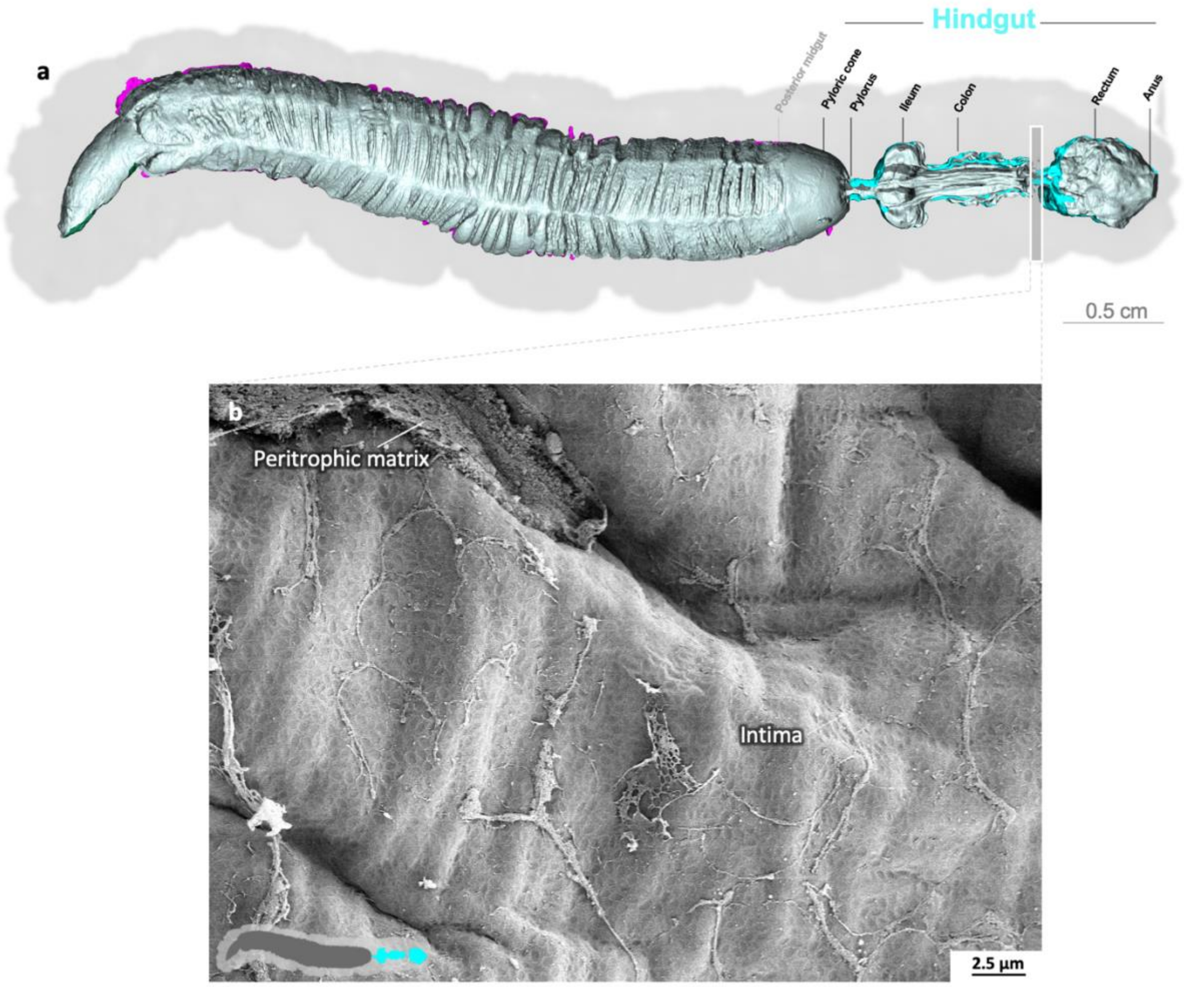

Figure S11: Hindgut (rectum), related to figure 11. In contrast to the more anterior hindgut parts, the rectum has a smooth intima. A micro-tomographic surface overview of the digestive system of *M. sexta* (**a**) shows the localization of the SEM insets (**b**-**e**).

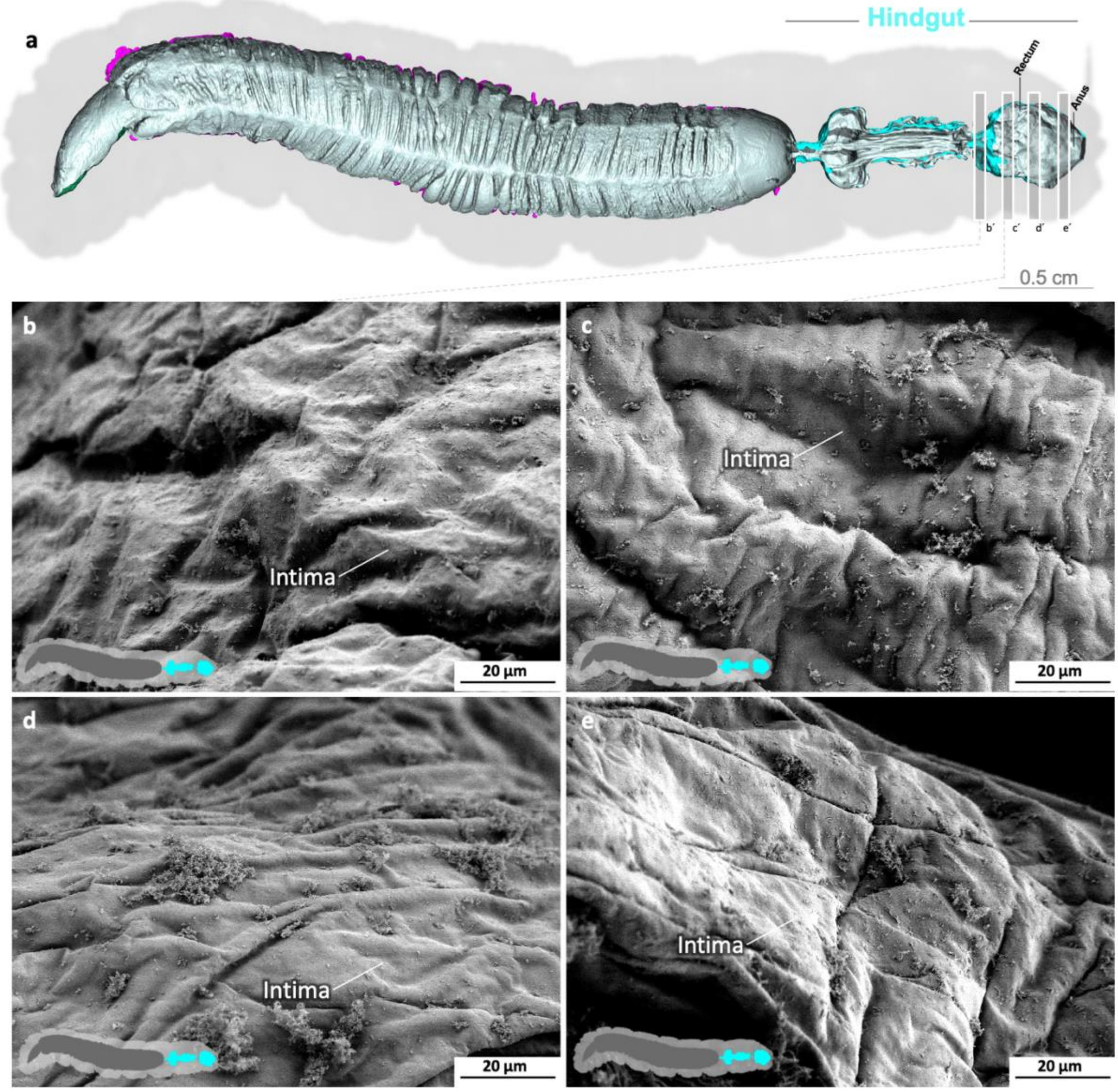

Supplement: Document S1. Figures S1–S11 [file mmc1.pdf]
